# Impact of IL6R genetic variants on treatment efficacy and toxicity response to sarilumab in rheumatoid arthritis

**DOI:** 10.1186/s13075-023-03209-1

**Published:** 2023-11-24

**Authors:** Luis Sainz, Pau Riera, Patricia Moya, Sara Bernal, Jordi Casademont, Cesar Díaz-Torné, Ana Milena Millán, Hye Sang Park, Adriana Lasa, Hector Corominas

**Affiliations:** 1 Rheumatology Department, Hospital de La Santa Creu I Sant Pau, Barcelona, Spain; 2 Department of Medicine, Universitat Autònoma de Barcelona (UAB), Barcelona, Spain; 3 Institut d’Investigació Biomèdica Sant Pau (IIB SANT PAU), Barcelona, Spain; 4 Pharmacy Department, Hospital de La Santa Creu I Sant Pau, Barcelona, Spain; 5 Biomedical Network Research Centre On Rare Diseases (CIBERER), Instituto de Salud Carlos III, Madrid, Spain; 6 Genetics Department, Institut d’Investigacions Biomèdiques Sant Pau - Hospital de La Santa Creu I Sant Pau, Barcelona, Spain; 7 Internal Medicine Department, Hospital de La Santa Creu I Sant Pau, Barcelona, Spain

**Keywords:** IL6R, Genetic variants, Sarilumab, Rheumatoid arthritis, Predictive factors

## Abstract

**Background:**

Sarilumab, an IL-6 receptor antagonist, is a first-line biologic disease-modifying anti-rheumatic drug for rheumatoid arthritis. The identification of genetic biomarkers as predictors of response to sarilumab could allow for a personalized treatment strategy to improve clinical outcomes.

**Methods:**

We conducted a retrospective cohort study of 62 patients treated with sarilumab to determine whether single-nucleotide polymorphisms (SNP) in the *IL6R* gene could predict efficacy and toxicity responses. Six SNPs previously described in the IL6R gene (rs12083537, rs11265618, rs4329505, rs2228145, rs4537545, and rs4845625) were genotyped in DNA samples obtained from these patients. Using parametric tests, we evaluated the association between these polymorphisms and clinicopathological features. Treatment response was assessed six months after treatment initiation. Satisfactory response was based on EULAR criteria. Low disease activity was determined according to DAS28 and CDAI and quantitative improvements in DAS28 and CDAI scores.

**Results:**

Three SNPs (rs4845625, rs4329505 and rs11265618) were significantly associated with response outcomes. All of the SNPs, except for rs12083537, had at least one significant association with dyslipidemia or hepatotoxicity.

**Conclusions:**

These findings support the potential clinical value of SNPs, particularly rs4845625, as potentially useful biomarkers to predict response to sarilumab in patients with RA.

## Introduction

Rheumatoid arthritis (RA) is a highly prevalent chronic systemic inflammatory disease. Clinically, RA is characterized by symmetrical peripheral polyarthritis with extraarticular manifestations [1]. The natural course of this disease may lead to progressive disability, systemic complications, and reduced quality of life [2, 3].

The principal aim of current therapeutic strategies is to achieve clinical remission or a state of low disease activity (LDA). If neither of these objectives are achieved, then the recommended approach is to adjust the treatment strategy, usually by switching to disease-modifying anti-rheumatic drugs (DMARD) [4]. In recent decades many highly effective biologic (bDMARD) and non-biologic therapies have been developed, leading to better disease control in many patients. Nonetheless, only limited progress has been made in identifying reliable biomarkers that could allow for personalized selection of the optimal DMARDs. At present, however, most of the available DMARDs lack robust supporting data [5]. Therefore, although the treatment selection process must take into account comorbidities and the costs of treatment, in most cases the treating physician can select treatment according to his or her professional experience, using a trial-and-error approach [6–8].

In recent years, interest in pharmacogenomics has grown due to its potential to explain much of the inter-individual differences in treatment response and predisposition to drug toxicity. The vast majority of studies published to date have focused on single nucleotide polymorphisms (SNP) as genetic biomarkers for methotrexate [9–12] and/or anti-TNF [13–19], and on new therapies, such as the IL-6R antagonist tocilizumab (TCZ) [20–26].

Sarilumab is a biologic disease-modifying antirheumatic drug (bDMARD) that exhibits specific binding affinity to both soluble and membrane-bound IL-6 receptors (IL-6R), thereby inhibiting IL-6 mediated signaling. In the last few years, this drug has received regulatory approval for the treatment of patients with moderately to severely active RA who are either intolerant or unresponsive to at least one conventional synthetic (cs)DMARD. Placebo-controlled clinical trials [27, 28] have reported response rates (ACR50 criteria) ranging from 40 to 45% at 6 months. Despite this notable efficacy, only 28–34% of patients in those studies achieved the therapeutic goal of DAS28-CRP (c-reactive protein) remission.

In this context, and in line with findings from pharmacogenetic studies on TCZ [20–26], we hypothesized that functional variations in the interleukin-6 receptor (IL6R) gene could affect treatment outcomes with sarilumab. Therefore, the aim of the present study was to assess whether genetic variants in the *IL6R* gene are associated with response to sarilumab and treatment-related toxicity in patients diagnosed with RA.

## Materials and methods

### Study population

We conducted a single-center, retrospective cohort study involving RA patients recruited from a tertiary referral hospital. Potential participants were identified through a search of pharmacy registries, in which we identified all patients who received sarilumab treatment between January 2018 and December 2021.

The inclusion criteria were as follows: confirmed diagnosis of RA based on the American College of Rheumatology /European League Against Rheumatism (EULAR) 2010 criteria [29]; prescription of sarilumab for RA; and age ≥ 18 years. Exclusion criteria were: presence of other rheumatic conditions (including connective tissue diseases or vasculitis) and loss of patient to follow-up.

Sociodemographic and clinical data were collected from electronical medical records. The following variables were assessed: age; sex; age at diagnosis; body mass index (BMI); previous treatments; comorbidities; baseline c-reactive protein (CRP) level; rheumatoid factor (RF) and anti-citrullinated protein antibody (ACPA) status; and sarilumab initiation and withdrawal dates.

The Disease Activity Score 28 (DAS28) and Clinical Disease Activity Index (CDAI) at treatment initiation and at 6-months were used to evaluate treatment response. Response was further evaluated based on the following parameters: satisfactory response (EULAR criteria); achievement of LDA, which was defined as a DAS28 score ≤ 3.2 or CDAI score ≤ 10, as well as quantifiable improvements in DAS28 and CDAI values at 6 months. Following EULAR guidelines, a satisfactory EULAR response was defined as a DAS28 improvement > 1.2 with a resulting DAS28 score ≤ 3.2. [30, 31].

Adverse effects (AE) were registered and classified by type [32] and by severity according to the Common Terminology Criteria for Adverse Events (CTCAE, v 6.0). AEs were further categorized into specific groups, as follows: hepatotoxicity, infections, hypersensitivity, gastrointestinal, hematological and dyslipidemia. To assess hepatotoxicity, hematological alterations, and dyslipidemia, we retrospectively reviewed the results of routine blood tests performed during follow-up every 6 months. Numeric values for transaminases, leukocytes, neutrophils, platelets, and lipids levels were recorded at the time any AE was detected.

The study protocol was approved by the respective institutional ethics committees and registered at clinicaltrials.gov (protocol code: IIBSP-IIL-2020–148). Prior to participation, all participants provided written informed consent for the collection of blood samples and subsequent genetic analyses.

### Genetic studies

Selection of the specific SNPs for analysis was based on the available literature and SNP functionality in the *IL6R* gene. The following SNPs were selected: rs12083537, rs11265618, rs4329505, rs2228145, rs4537545, and rs4845625 (Table 1). The rationale behind the selection of these SNPs has been described elsewhere [20–24, 26, 33]. Importantly, all of the chosen SNPs have a minor allele frequency (MAF) > 0.10 in the European population according to the Allele Frequency Aggregator (ALFA) [34].

**Table 1:** Selected functional polymorphisms in the *IL6R* gene

**refSeg**	**Genomic position** (GRCh38)	**MAF**	**Alleles**
rs12083537	chr1:154408627	0.21	A > **G**
rs11265618	chr1:154457616	0.17	C > **T**
rs4329505	chr1:154459944	0.17	T > **C**
rs2228145	chr1:154454494	0.40	A > **C**
rs4537545	chr1:154446403	0.41	C > **T**
rs4845625	chr1:154449591	0.43	**T** > C

The minor allele is highlighted in bold

*Abbreviations*: *IL6R* Interleukin 6 Receptor, *MAF* Minor allele frequency reported in the European population

Genomic DNA was automatically extracted from peripheral whole-blood samples using the Autopure LS system (Qiagen; Hilden, Germany). Genotyping of the selected SNPs was performed through real-time PCR using TaqMan® SNP genotyping assays (Applied Biosystems; Foster City, CA, USA). All cases were successfully genotyped.

### Statistical analyses

The Hardy–Weinberg equilibrium for all SNPs was assessed with the chi-square test. Associations between the SNPs and treatment outcome variables were examined considering various models of inheritance—including codominant, dominant, and recessive models—as appropriate.

Quantitative data are presented as means (standard deviation [SD]) for normally distributed variables. The Shapiro–Wilks test was applied to assess distribution normality. Student’s T test or ANOVA was employed for normally distributed variables, depending on the number of groups being compared. For qualitative dichotomous variables, bivariate associations were evaluated with Pearson’s chi-square (χ2) or Fisher’s exact test. Associations between the SNPs and qualitative response variables were tested using χ2 tests.

All statistical tests were two-sided, with the cut-off for statistical significance set at 5% (α = 0.05). The IBM SPSS Statistics for Windows, v. 26.0 (IBM Corp. Armonk, NY, USA: IBM Corp) was used to perform all statistical analyses.

## Results

### Patient population

A total of 62 patients were included in the study. Table 2 summarizes the baseline demographic and clinical characteristics of these patients. The mean (SD) disease duration at initiation of sarilumab was 17.1 (10.9) years. At the end of the data collection period (at six months of follow-up), one-third of the patients (20/62, 32.3%) were still actively receiving sarilumab.

**Table 2:** Baseline patient characteristics (*n* = 62)

Variables	n (%)	Mean (SD)
Female / Male	55 (88.7) / 7 (11.3)	
Age at diagnosis, years		48.1 (14.9)
Disease duration, years		17.1 (10.9)
Erosive RA	36 (58.1)	
RF positive	46 (74.2)	
ACPA positive	42 (67.8)	
Smoking habit		
Non-smoker	36 (58.1)	
Ex-smoker	11 (17.7)	
Smoker	6 (9.7)	
Missing data	9 (14.5)	
Body mass index		28.8 (6.3)
Number of previous cDMARDs		2.5 (1.3)
Number of previous bDMARDs		2.8 (2.2)
Duration of treatment, months		18.2 (14.4)
Baseline DAS28		5.3 (1.2)
Baseline CDAI		24.9 (9.8)

*Abbreviations*: *RA* Rheumatoid arthritis, *ACPA* Anticitrullinated protein antibodies, *DAS28* Disease Activity Score including 28 joints, *bDMARD* biological disease-modifying antirheumatic drug, *cDMARD* conventional biological disease-modifying antirheumatic drug, *CDAI* Clinical Disease Activity Index, *RF* Rheumatoid factor

Effectiveness.

Of the 62 patients initially included in the study, seven (11.3%) were excluded from the efficacy analysis due to premature treatment discontinuation caused by early toxicity (6 patients) or because the results of the disease activity assessment were not recorded in the electronic medical records (one patient).

At the 6-month follow up, the mean (SD) decrease in DAS28 and CDAI scores was 2.6 (1.3) and 10.6 (9.3), respectively. A satisfactory EULAR response rate was achieved in 76.4% of cases. LDA for DAS28 and CDAI was achieved in 68.5% and 63.5% of patients, respectively. The mean (SD) duration of treatment was 18.2 months (14.4).

No significant differences in treatment response variables were observed according to sex, seropositivity, number of previous cDMARDs or bDMARDs, age at diagnosis, or BMI. Although ex-smokers (*n* = 11) and current smokers (*n* = 5) had a better response than never-smokers (*n* = 36) in terms of achieving.

DAS28-LDA (90.9% and 100% vs 58.3%, *p* = 0.02), no differences in CDAI-

LDA rates were found.

### Adverse events

Table 3 summarizes the frequency and severity of AEs for the 62 patients. All AEs were mild or moderate; no severe AEs were reported. The two most common types of AE were hematological alterations and dyslipidemia. Of the 24 hematological AEs registered, 11 were mild and 13 moderate. In most cases, these were managed by observation (*n* = 9), temporary discontinuation of sarilumab (*n* = 3), or dose reduction (*n* = 7). The most common hematological AE was neutropenia (22 cases), with a median (SD) value of 1.06 × 10^9^/L (360). In terms of dyslipidemia, the median (SD) total cholesterol value was 284.6 (43.9) mg/dL. Only three patients required initiation of cholesterol lowering therapy due to sarilumab.

**Table 3:** Sarilumab-related adverse events (62 patients)

**Adverse event**	**n** (**%**)	**Mild, n (%)**	**Moderate, n (%)**	**Sarilumab discontinuation n (%)**
**Hepatotoxicity**	6 (9.7)	5 (8.1)	1 (1.6)	0
**Infections**	4 (6.5)	1 (1.6)	3 (4.9)	0
**Hypersensitivity/Intolerance**	8 (12.9)	2 (3.2)	6 (9.7)	7 (11.9)
**Gastrointestinal**	0 (0)	0 (0)	0 (0)	0
**Hematological, total** Neutropenia (*n* = 22) Thrombocytopenia (*n* = 2)	24 (38.7)	11 (17.7)	13 (20.9)	5 (8.1)
**Dyslipidemia**	18 (29)			

### Genetic determinants and response to treatment

The genotypic frequencies of all six SNPs were in Hardy–Weinberg equilibrium. A linked inheritance was observed between rs4329505 and rs11265618, as all individuals carrying the T allele for rs4329505 also had the C allele for rs11265618, and vice versa. Consequently, the results for both of those SNPs were analogous.

**Fig. 1:**
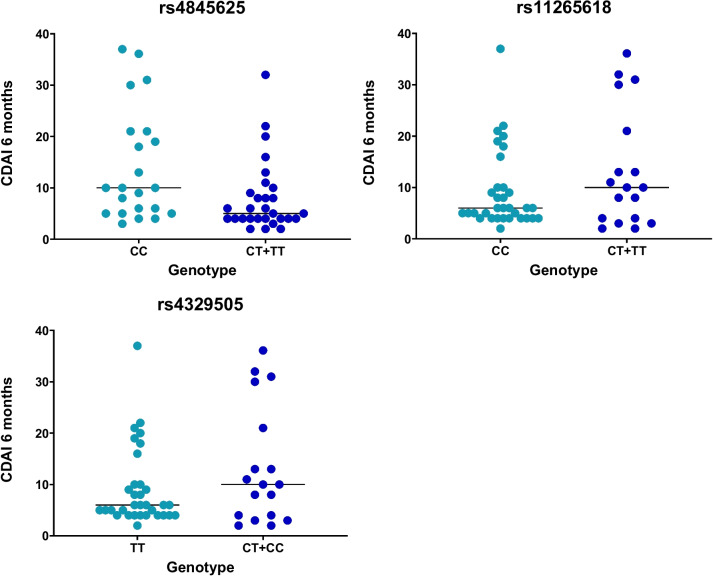
Dot plot of CDAI at 6 months for genotypes of rs4845625, rs11265618, and rs4329505. The line at each genotype represents the median. The consideration of CDAI-LDA at 6 months was considered when the CDAI score was equal or under 10. Abbreviations: CDAI: Clinical Disease Activity Index; LDA: Low Disease Activity

On the univariate analysis, three SNPs (rs4845625, rs4329505, rs11265618) were significantly associated with response outcomes (Table 4). In Fig. 1, the dot plot graphs for CDAI at 6 months, which is used to determine the CDAI-LDA, for these three SNPs are shown.

**Table 4:** Associations between the six SNPs and response outcomes at 6 months

SNPs	Genotype (n)	DAS28 improvement			DAS28-LDA			CDAI improvement			CDAI-LDA		
		**Mean absolute value**	**Genetic model**	***p***	**%**	**Genetic model**	***p***	**Mean absolute value**	**Genetic model**	***p***	**%**	**Genetic model**	***p***
rs4845625	T/T (9)	2.79	Cod	0.55	71.4	Cod	0.09	13.36	Cod	0.13	71.4	Cod	0.06
	C/T (28)	2.82	Rec	0.28	82.6	Rec	**0.037**	17.69	Rec	0.06	78.3	Rec	**0.02**
	C/C (25)	2.34			52.4			10.69			45.5		
rs11265618	C/C (42)	2.91	Cod	0.08	73.5	Cod	0.12	15.64	Cod	0.31	73.5	Cod	0.08
	C/T (17)	1.87	Dom	**0.048**	50	Dom	0.29	10.12	Dom	0.18	40	Dom	**0.039**
	T/T (3)	2.82			100			15.66			66.7		
rs4329505	T/T (42)	2.91	Cod	0.08	73.5	Cod	0.12	15.64	Cod	0.31	73.5	Cod	0.08
	C/T (17)	1.87	Dom	**0.048**	50	Dom	0.29	10.12	Dom	0.18	40	Dom	**0.039**
	C/C (3)	2.82			100			15.66			66.7		
rs12083537	A/A (40)	2.36	Cod	0.21	72.7	Cod	0.26	13.37	Cod	0.48	63.6	Cod	0.35
	A/G (20)	3.13	Dom	0.10	64.7	Dom	0.39	16.05	Dom	0.56	66.7	Dom	0.97
	G/G (2)	2.05			0			3			0		
rs2228145	A/A (22)	2.36	Cod	0.62	73.7	Cod	0.82	13.39	Cod	0.95	63.2	Cod	0.99
	C/A (27)	2.71	Dom	0.35	66.7	Dom	0.55	14.49	Dom	0.75	63.6	Dom	0.97
	C/C (13)	2.87			63.6			14.41			63.6		
rs4537545	C/C (20)	2.31	Cod	0.50	72.7	Cod	0.92	13.27	Cod	0.91	61.1	Cod	0.95
	C/T (28)	2.68	Dom	0.29	66.7	Dom	0.68	14.06	Dom	0.73	63.6	Dom	0.79
	T/T (14)	2.96			66.7			15.23			66.7		

*Abbreviations*: *CDAI* Clinical Disease Activity Index, *Cod* Codominant, *DAS28* Disease Activity Score including 28 joints, *Dom* Dominant, *p* p-value, *Rec* Recessive, *SNPs* Single Nucleotide Polymorphisms

Patients carrying the CC genotype for rs4845625 had worse response rates to sarilumab as measured by CDAI and DAS28 LDA rates (45.5% and 52.4% vs. 76.7% and 80% in the CT + TT genotypes, respectively; *p* = 0.021 and *p* = 0.037). CC carriers showed less improvement in DAS28 (2.34 vs. 2.8, *p* = 0.27) and CDAI (10.7 vs. 16.7, *p* = 0.066), although these differences were not statistically significant. These findings are consistent with a recessive genetic model.

For rs4329505 and rs11265618, the genetic model that best fit the data was the dominant model, as patients homozygous for the wild-type allele (TT for rs4329505 and CC for rs11265618) showed better remission rates than the other patients; specifically, remission rates (CDAI-LDA) were 73.5% vs. 44.4% (*p* = 0.039) and the quantitative improvement in DAS28 was 2.9 vs. 2.0 (*p* = 0.048). No significant differences were found for DAS28 LDA, CDAI improvement, and/or EULAR response rates.

No statistically significant associations were observed for the remaining three SNPs (rs12083537, rs2228145, rs4537545).

### Genetic determinants and adverse effects

On the univariate analysis, all SNPs (except for rs12083537) were significantly associated with dyslipidemia and/or hepatotoxicity (Table 5). None of the SNPs showed a statistically significant association with any of the other AEs observed during the study period.

**Table 5:** Associations between the SNPs and hepatotoxicity and dyslipidemia

SNPs	Genotype (n)	Hepatotoxicity			Dyslipidemia		
		**%**	**Genetic model**	***p***	**%**	**Genetic model**	***p***
rs4845625	T/T (9)	33.3	Cod	0.07	44.4	Cod	0.14
	C/T (28)	7.1	Dom	**0.027**	35.7	Rec	0.056
	C/C (25)	4			16		
rs11265618	T/T (42)	14.3	Cod	0.08	23.8	Cod	0.41
	C/T (17)	0	Dom	**0.026**	41.2	Dom	0.19
	C/C (3)	0			33.3		
rs4329505	T/T (42)	14.3	Cod	0.08	23.8	Cod	0.41
	C/T (17)	0	Dom	**0.026**	41.2	Dom	0.19
	C/C (3)	0			33.3		
rs2228145	A/A (22)	13.6	Cod	0.75	50	Cod	0.028
	C/A (27)	7.4	Dom	0.44	5	Dom	**0.008**
	C/C (13)	7.7			2		
rs4537545	C/C (20)	15	Cod	0.64	50	Cod	**0.046**
	C/T (28)	7.1	Dom	0.34	17.9	Dom	**0.014**
	*T/T (14)*	*7.1*			*21.4*		

*Abbreviations*: *SNPs* Single Nucleotide Polymorphisms, *p* p-value

Compared to AC + CC carriers, patients carrying the AA genotype for rs2228145 had a higher incidence of dyslipidemia (50% vs. 17.5%, *p* = 0.008). Similarly, for rs4537545, the incidence of dyslipidemia was greater in CC carriers than for CT + TT genotypes (50% vs. 19%, *p* = 0.014).

Both rs4329505 and rs11265618 were significantly associated with hepatotoxicity. More specifically, TT carriers for rs4329505 and CC carriers for rs11265618 had higher rates of hepatotoxicity compared to the other genotypes (14.3% vs. 0%, *p* = 0.026).

Patients with the TT genotype for rs4845625 had a significantly higher incidence of hepatotoxicity compared to carriers of the C allele (CT + CC) (33.3% vs. 5.7%, *p* = 0.03). We also observed a trend towards an association between rs4845625 and dyslipidemia, as evidenced by the higher incidence of dyslipidemia for patients carrying the T allele (TT + CT); however, this association was not statistically significant.

## Discussion

This study was conducted to investigate possible associations between six different SNPs in the *IL6R* gene and treatment response outcomes in patients with rheumatoid arthritis treated with sarilumab over a 6-month period. To our knowledge, this is the first study to examine the pharmacogenetics of sarilumab. Significant associations were identified between three SNPs (rs4845625, rs4329505, rs11265618) and certain response outcomes (DAS28 improvement, DAS28-LDA, CDAI-LDA). Similarly, five of the six SNPs (rs2228145, rs4329505, rs11265618, rs4537545, rs4845625) were significantly associated with adverse events (hepatotoxicity and dyslipidemia).

Our findings with regard to the rs4845625 SNP are particularly noteworthy given how consistent these results were. This SNP was positively associated with LDA rates for both DAS28 and CDAI. Interestingly, there were clinically-relevant differences between the groups, with a 20–30% gap between them in terms of achieving the treatment aims (remission or LDA) (Table 4). The rs4845625 SNP is located in Chr1 (q21.3) g.154422067, in intron 7 of the *IL6R* gene. In silico tools such as ESE Finder predict that this SNP may alter the splicing process, thus leading to changes in the amount or functionality of the resulting protein. These changes may result in differences in treatment responses, as previous studies have indicated that RA patients with higher concentrations of soluble IL-6R tend to show poorer responses to TCZ [35]. Furthermore, it has been demonstrated that other SNPs (such as rs2228145) in IL6R can influence the balance between membrane-bound IL-6R and soluble IL-6R [36].

Previous studies have shown several associations between rs4845625 and inflammatory pathways, including differences in plasma IL-6 levels [37] and basal levels of inflammatory markers in healthy cohorts [33]. Our research group was the first to report the association between rs4845625 and differences in response outcomes in patients with RA treated with tocilizumab, another IL-6 receptor antagonist [20]. In that study, patients with the CC genotype had a lower response to TCZ (as measured by DAS28-LDA) than patients with the CT + TT genotypes (58.3% vs. 82.4%, respectively). Those findings are consistent with the results of the present study with sarilumab. Moreover, the results of the present study provide further support for our earlier results with TCZ, as we have now identified the same association with another IL-6R antagonist (sarilumab), suggesting that genetic variations in *IL6R* can modulate treatment response. Additionally, in the present study we utilized more reliable and consistent response outcome measures, including the addition of CDAI, which is not influenced by the effect of IL-6 inhibitors on C-reactive protein [38].

Both the rs4329505 and rs11265618 SNPs exhibited similar results due to linked inheritance, which makes it challenging to identify the specific genetic changes that truly modulate treatment response. Apart from a substantial 30% variation in treatment response (CDAI-LDA), these SNPs were the only ones to demonstrate significant differences in terms of the quantitative reduction in DAS28. Enevold et al. [23] evaluated treatment response outcomes (number of swollen joints) to TCZ in patients with RA, finding similar differences for rs4329505. In that study, allele C carriers (CC + CT) also had a lower response to treatment. In our previous study with TCZ [20], we also found a trend toward better response rates (measured by DAS28 and EULAR) in patients with the TT genotype, although these differences were not statistically significant. By contrast, rs4329505 has been investigated in two retrospective studies by Maldonado-Montoro et al. [22] and Luxembourger et al. [24], without finding significant associations with response rates.

In our previous study with TCZ, we found no significant association between rs11265618 and response outcomes. By contrast, in the present study with sarilumab, we did observe a significant association, a finding that is in line with the study by Maldonado-Montoro et al. [21], who described an association with the CC genotype for rs11265618, indicating a better response in terms of LDA after 12 months of TCZ therapy. Previous studies of IL-6 inhibitors have not found consistent results for either rs4329505 or rs11265618 [21, 24]. Although our findings provide support for a coherent trend with regards to the value of those SNPs as potential biomarkers, further confirmation is warranted before they can be considered reliable biomarkers for IL-6 inhibitor therapy.

Most of the AEs reported in the present series were mild or moderate, with no severe AEs detected during follow-up (mean, 18 months). The most commonly reported AEs were hematological, but they rarely required discontinuation of sarilumab (8.1%). Additionally, we found no associations between these AEs and any genetic determinants.

Although we found a positive association between several AEs with at least one SNP, in most cases the AEs were not clinically relevant and thus did not require a therapeutic change. With regards to hepatotoxicity, given that these cases were only of mild to moderate severity and also relatively rare (9.7%), we believe that the observed associations were only incidental findings. For dyslipidemia, we found significant associations for rs2228145, with higher rates in AA carriers, and for rs4537545, with higher rates in CC carriers. Although the clinical significance of these associations may be low, as dyslipidemia secondary to TCZ treatment has not been linked to an increased risk of cardiovascular events [39–43], the interplay between lipid profiles and inflammatory pathways, such as IL-6, has been extensively described [42, 44–46]. Therefore, it is plausible that alterations in the *IL6R* pathway may influence the development of dyslipidemia. In our previous study with TCZ, we observed that patients carrying the CT + TT genotype had a better response to treatment but a higher incidence of dyslipidemia. In the present study with sarilumab, we have found similar results in terms of treatment efficacy. Although we found no significant association between genotype and dyslipidemia, we did observe a trend towards a higher incidence of dyslipidemia for TT + CT genotypes than for CC genotypes (33.3% vs. 16%, *p* = 0.056), a finding that is consistent with the results of our previous study.

This study has several limitations that should be considered when interpreting the results. First, the retrospective design and the relatively small sample size may have limited our ability to identify a significant association between some SNPs and response outcomes, particularly in those SNPs (rs11265618 and rs4329505) with a low frequency of the mutated allele. Nonetheless, the homogeneity of this cohort and the fact that our results were comparable to those described in our previous study with TCZ, strengthen the validity of our findings. Second, we only evaluated the six most promising *IL6R* SNPs; other genetic variants not included in the present study could also be predictor of response to sarilumab. Another strength is that this is the first pharmacogenetic study of sarilumab. Moreover, since sarilumab is tipically used in current clinical practice after failure of at least one other bDMARD, this is the first study to provide data on this unique cohort.

The pharmacogenomics of RA is highly complex and further research is needed to obtain more robust, clinically-relevant evidence to determine the association between genetic markers and treatment response in patients with RA. However, based on our findings, we believe that rs4845625 may be a useful biomarker to help predict treatment response. However, more comprehensive research is needed before it can be considered for use in routine clinical practice.

## Conclusions

The results of this study suggest that several *IL6R* polymorphisms—rs4845625, rs4329505 and rs11265618—may be associated with treatment response as measured by CDAI and DAS28 LDA rates and absolute improvement in DAS28 after six months of sarilumab in patients with RA. In addition, we have also shown that five SNPs (rs2228145, rs4329505, rs11265618, rs4537545, rs4845625) are associated with the development of certain adverse events—particularly hepatotoxicity and dyslipidemia—during the follow-up, even though they showed limited clinical significance. To our knowledge, these associations have not been previously reported.

## Data Availability

The datasets generated and analysed during the current study are not publicly available due to containing clinical information which could constitute potential patient identifiers, but are available from the corresponding author on reasonable request.

## Abbreviations

ACPA: Anti-citrullinated protein antibody
AE: Adverse effects
CDAI: Clinical disease activity index
CRP: C-reactive protein
DAS28: Disease activity score 28
bDMARD: Biologic Disease-Modifying anti-rheumatic drugs
DMARD: Disease-Modifying anti-rheumatic drugs
EULAR: European league against rheumatism
IL-6R: IL-6 receptors
LDA: Low disease activity
MAF: Minor allele frequency
RA: Rheumatoid arthritis
RF: Rheumatoid factor
SD: Standard deviation
SNP: Single nucleotide polymorphism
TCZ: Tocilizumab
